# Cytokine Combinations wERKing to a Different Tune

**DOI:** 10.1097/HS9.0000000000000623

**Published:** 2021-07-30

**Authors:** David G. Kent

**Affiliations:** York Biomedical Research Institute, Department of Biology, University of York, United Kingdom

Researchers studying the hematopoietic system regularly inherit protocols with growth factor combinations and concentrations whose origins can be traced back to papers in the 1980s and 1990s during the heyday of making and testing recombinant cytokines. This was a time of trial and error with a lot of “low,” “medium,” and “high” concentrations being tested for their relative effects on hematopoietic colony formation. Systematic efforts to combine different cytokines in multifactorial studies dotted the literature in the early 2000s and companies leading the field in the production of media supporting hematopoietic cell growth adopted core formulations that still stand today.^1,2^ Fast forward 20 years and the collection of researchers using these same formulations numbers in the thousands and, aside from an occasional addition of factor “X” to test molecules/compounds 1 by 1 for their effect on hematopoietic cell growth, these core formulations remain largely unaltered. As a result, the components of these recipes are often thought of as a binary presence or absence (ie, with or without erythropoietin, stem cell factor, interleukin-7, etc.) rather than a complicated and dynamic set of signals with potential antagonistic and synergistic effects. A recent article published in *Blood* from Timm Schroeder’s group sheds light on how different cytokine combinations can elicit distinct signaling dynamics in human hematopoietic stem cells using extracellular signal-regulated kinase (ERK) signaling (also known as mitogen-activated protein kinase signaling and a main mediator of cellular proliferation) as a model system and implores us all to think beyond a simple on/off system for cytokine signaling.^3^

**Figure:**
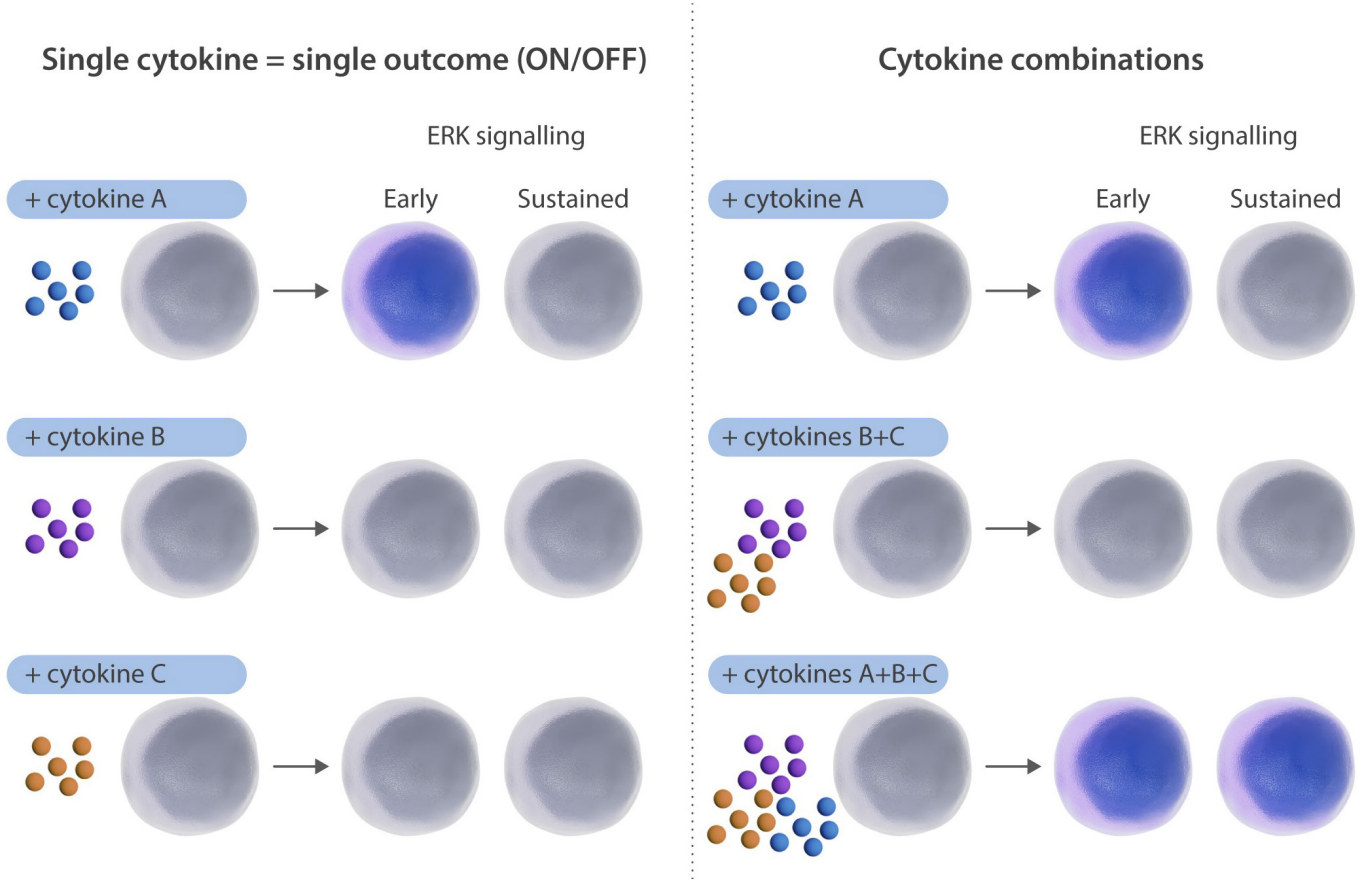


## ERK dynamics are altered by cytokine combinations

Perhaps the most exciting thing about the Wang et al^3^ article is that it shows that ERK signaling dynamics were intimately linked with eventual cell fate, meaning that snapshot measurements of active signaling are not fully able to predict cellular outcome. Using a sophisticated microfluidic live cell culturing device in combination with an ERK kinase translocation reporter (ERKKTR), the authors were able to chart different ERK dynamics caused by different levels and combinations of cytokines. Importantly, these differences in ERK dynamics were not predictable by the current collection of cell surface markers used to stratify hematopoietic stem and progenitor cells but were consistently associated with cells that became one cell type versus another. Exemplifying this, the levels of CD45RA (an early marker of hematopoietic stem cell differentiation) were higher in the progeny of hematopoietic stem cells with intermediate levels of ERK signaling compared with those with transient or sustained ERK signaling, suggesting that these cells were less likely to be retained in the immature state.

## Monitoring molecular dynamics in live cells

Using biosensors to measure real-time cell outputs is a rapidly growing area of research, although the tools to measure specific molecules are often quite labor intensive to develop and restrict more global screening type approaches. For example, the ERKKTR reporter used in the study by Wang et al^3^ was its own *Cell* article in 2014^4^ and cannot give any insight into any of the complementary cell signaling pathways that may be operative in the cell(s) of interest. Approaches where the sensors themselves are more modular and flexible might be explored as has already been done for monitoring the secretion of cytokines from live cells.^5^ The obvious challenge will be making a modular sensor or multifactorial set of sensors within the cell which would revolutionize our ability to study signaling in live cells with linked functional outcome.

Together, this data from the Schroeder lab emphasizes the importance of pushing the technical boundaries of our “established” systems to uncover new knowledge that incorporates dynamic measurements in the context of assessing cell function. The recent explosion of static measurements of transcriptomes, genomes, epigenomes, and proteomes, etc. has produced a truly incredible data bank to draw from, but this article clearly makes a case for pushing beyond these static measurements to ensure that the complexity of systems such as cytokine signaling is able to be studied in a real-time and dynamic manner. These sorts of tools are likely to challenge some long-standing protocols and beliefs about the nature of cytokine signaling and the heterogeneity in strength and dynamics that will be an incredibly interesting space to watch in coming years.

## Disclosures

The author has no conflicts of interest to declare.
